# Safety and Efficacy of 5 Years of Treatment With Recombinant Human Parathyroid Hormone in Adults With Hypoparathyroidism

**DOI:** 10.1210/jc.2019-01010

**Published:** 2019-08-01

**Authors:** Michael Mannstadt, Bart L Clarke, John P Bilezikian, Henry Bone, Douglas Denham, Michael A Levine, Munro Peacock, Jeffrey Rothman, Dolores M Shoback, Mark L Warren, Nelson B Watts, Hak-Myung Lee, Nicole Sherry, Tamara J Vokes

**Affiliations:** 1 Endocrine Unit, Massachusetts General Hospital and Harvard Medical School, Boston, Massachusetts; 2 Division of Endocrinology, Diabetes, Metabolism, and Nutrition, Mayo Clinic, Rochester, Minnesota; 3 Division of Endocrinology, College of Physicians and Surgeons, Columbia University, New York, New York; 4 Michigan Bone and Mineral Clinic, PC, Detroit, Michigan; 5 Clinical Trials of Texas, Inc., San Antonio, Texas; 6 Division of Endocrinology and Diabetes and Center for Bone Health, Children’s Hospital of Philadelphia, Philadelphia, Pennsylvania; 7 Department of Medicine, Division of Endocrinology, Indiana University School of Medicine, Indianapolis, Indiana; 8 University Physicians Group – Research Division, Staten Island, New York; 9 Endocrine Research Unit, Department of Medicine, San Francisco Veterans Affairs Medical Center, University of California, San Francisco, California; 10 Endocrinology and Metabolism, Physicians East, Greenville, North Carolina; 11 Osteoporosis and Bone Health Services, Mercy Health, Cincinnati, Ohio; 12 Shire Human Genetic Therapies, Inc., a member of the Takeda group of companies, Lexington, Massachusetts; 13 Shire Human Genetic Therapies, Inc., a member of the Takeda group of companies, Cambridge, Massachusetts; 14 Section of Endocrinology, University of Chicago Medicine, Chicago, Illinois

## Abstract

**Context:**

Conventional hypoparathyroidism treatment with oral calcium and active vitamin D is aimed at correcting hypocalcemia but does not address other physiologic defects caused by PTH deficiency.

**Objective:**

To evaluate long-term safety and tolerability of recombinant human PTH (1-84) [rhPTH(1-84)].

**Design:**

Open-label extension study; 5-year interim analysis.

**Setting:**

12 US centers.

**Patients:**

Adults (N = 49) with chronic hypoparathyroidism.

**Intervention(s):**

rhPTH(1-84) 25 or 50 µg/d initially, with 25-µg adjustments permitted to a 100 µg/d maximum.

**Main Outcome Measure(s):**

Safety parameters; composite efficacy outcome was the proportion of patients with ≥50% reduction in oral calcium (or ≤500 mg/d) and calcitriol (or ≤0.25 µg/d) doses, and albumin-corrected serum calcium normalized or maintained compared with baseline, not exceeding upper limit of normal.

**Results:**

Forty patients completed 60 months of treatment. Mean albumin-corrected serum calcium levels remained between 8.2 and 8.7 mg/dL. Between baseline and month 60, levels ± SD of urinary calcium, serum phosphorus, and calcium-phosphorus product decreased by 101.2 ± 236.24 mg/24 hours, 1.0 ± 0.78 mg/dL, and 8.5 ± 8.29 mg^2^/dL^2^, respectively. Serum creatinine level and estimated glomerular filtration rate were unchanged. Treatment-emergent adverse events (AEs) were reported in 48 patients (98.0%; hypocalcemia, 36.7%; muscle spasms, 32.7%; paresthesia, 30.6%; sinusitis, 30.6%; nausea, 30.6%) and serious AEs in 13 (26.5%). At month 60, 28 patients (70.0%) achieved the composite efficacy outcome. Bone turnover markers increased, peaked at ∼12 months, and then declined to values that remained above baseline.

**Conclusion:**

Treatment with rhPTH(1-84) for 5 years demonstrated a safety profile consistent with previous studies and improved key biochemical parameters.

## Teaser

Treatment of hypoparathyroidism rhPTH(1-84) for 5 years had a safety profile consistent with other studies and improved key biochemical parameters, including normalization of urinary calcium level.

PTH plays a central role in mineral homeostasis by stimulating renal reabsorption of calcium, promoting renal phosphate excretion, and stimulating conversion of 25-hydroxyvitamin D to 1,25-dihydroxyvitamin D (1,25[OH]_2_D), the fully active form of vitamin D. Moreover, 1,25[OH]_2_D enhances absorption of calcium and phosphate from the gastrointestinal tract (1). In addition, PTH is a potent regulator of bone turnover (*i.e.,* the coupled process of bone resorption and bone formation) (2–4); deficiency of PTH results in decreased bone turnover (2, 4). Conventional therapy for hypoparathyroidism includes oral calcium and active vitamin D (*e.g.,* calcitriol), as well as thiazide diuretics and magnesium supplementation as needed. Although this approach can correct the hypocalcemia associated with hypoparathyroidism, it does not replace other functions of PTH and can lead to or worsen hypercalciuria (2, 5). Other concerns with conventional therapy include unpredictable episodes of hypocalcemia and hypercalcemia; increased serum calcium-phosphorus (Ca × P) product; and complications such as extraskeletal calcifications, nephrolithiasis, nephrocalcinosis, and decreased kidney function (2, 5–7). Reduced quality of life and symptoms of hypocalcemia are consistent findings among patients with hypoparathyroidism receiving conventional treatment (8–13).

Recombinant human PTH [rhPTH(1-84)] is full-length PTH that is approved in the United States and Europe as an adjunctive treatment of adults with hypoparathyroidism (14, 15). Safety and efficacy of rhPTH(1-84) in patients with hypoparathyroidism have been demonstrated in short-term, placebo-controlled and open-label studies (16–20). In the pivotal REPLACE (Use of NPSP558 in the Treatment of Hypoparathyroidism) Study, a 24-week, double-blind, placebo-controlled, randomized phase 3 study conducted with 134 patients, 53% of patients receiving rhPTH(1-84) vs 2% of patients receiving placebo met the primary study end point (≥50% reduction from baseline in oral calcium and calcitriol doses with maintenance of normal albumin-corrected serum calcium) (17). In addition, patients receiving rhPTH(1-84) in REPLACE showed significant reductions in serum phosphorus throughout the 24-week study compared with patients receiving placebo (21). In both study groups, urinary calcium excretion declined from baseline, but at week 24 in both groups, the mean urinary calcium excretion remained above the upper limit of normal (ULN) for women and near the ULN for men (17, 21).

RACE (Open-label Study Investigating the Safety and Tolerability of NPSP558, a Recombinant Human Parathyroid Hormone [rhPTH(1-84)], for the Treatment of Adults With Hypoparathyroidism—A Clinical Extension Study; ClinicalTrials.gov identifier, NCT01297309) was an open-label extension study designed to assess long-term safety and efficacy of rhPTH(1-84) in patients with hypoparathyroidism. This article reports 5-year safety and efficacy of treatment with rhPTH(1-84) and examines changes in mineral homeostasis, kidney function, and skeletal parameters.

## Materials and Methods

### Study design and patients

**Figure 1.**
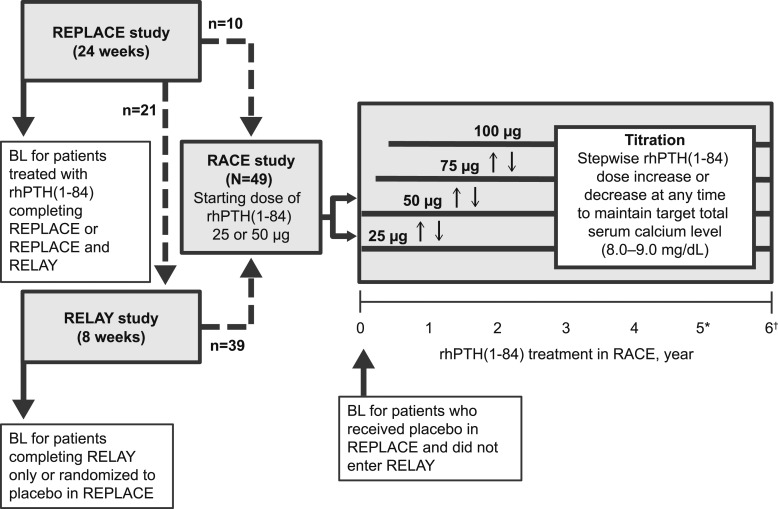
Study design. Dotted lines indicate that treatment interruptions could have occurred between studies. Baseline values were those measured just before initiation of rhPTH(1-84) treatment, whether that occurred at the start of REPLACE, RELAY, or RACE. BL, baseline; *Current analysis; †end of study.

RACE was an open-label extension conducted at 12 centers in the United States. The primary objective was to assess long-term safety and tolerability of rhPTH(1-84) in adult patients with hypoparathyroidism (Fig. 1). Patients were eligible to participate in RACE if they completed the randomized, placebo-controlled, 24-week REPLACE Study and/or the 8-week open-label, dose-blind RELAY (Study of Safety and Efficacy of rhPTH[1-84] of Fixed Doses of 25 and 50 μg in Adults With Hypoparathyroidism) Study. A treatment interruption between the end of treatment in REPLACE or RELAY and enrollment in RACE was permitted. Additional eligibility criteria included serum creatinine level <1.5 mg/dL (<132.6 µmol/L) at enrollment, total serum calcium level at or below the ULN based on local laboratory result before enrollment, and serum 25(OH)D ≤1.5 times the ULN (100 ng/mL) within 16 weeks before enrollment. Patients were permitted to continue taking most baseline concomitant medications during the trial, including thiazide diuretics, hormone therapy (estrogen with or without progesterone), and antihypertensives. Other drugs known to affect bone or mineral metabolism were prohibited (*e.g.*, calcitonin, cinacalcet, raloxifene, bisphosphonates, fluoride).

Investigators were permitted to introduce thiazide diuretics for control of hypercalciuria at or after week 16 for patients who were on a stable dose of rhPTH(1-84), had a 24-hour urine calcium level of >300 mg (>7.5 mmol) for men or >250 mg (>6.25 mmol) for women, and had not been receiving diuretics before the start of the study. Investigators administered sufficient supplemental cholecalciferol and/or ergocalciferol during the study to maintain patients’ serum 25(OH)D levels between 30 and 100 ng/mL.

Patients self-administered rhPTH(1-84) subcutaneously, once daily, in the morning. For this study, a starting dose of 50 µg/d was used for patients with a total serum calcium concentration ≤9.5 mg/dL (≤2.37 mmol/L) and for patients with a serum calcium concentration >9.5 mg/dL who were taking ≥500 mg of calcium and/or any calcitriol. A starting dose of 25 µg/d was used for patients with a total serum calcium concentration >9.5 mg/dL who were taking <500 mg of supplemental calcium and no calcitriol. The dose of rhPTH(1-84) could be increased or decreased by the investigator in stepwise increments of 25 µg up to a maximum dose of 100 µg/d any time during the study, with the goal of achieving or maintaining total serum calcium levels in the target range of 8.0 to 9.0 mg/dL (2.00 to 2.25 mmol/L; Fig. 1). Patients continued taking the dose of rhPTH(1-84) at which their serum calcium levels were stable in the target range and that permitted the lowest possible doses of oral calcium and calcitriol.

The study was conducted in accordance with applicable International Council for Harmonization Guidelines, Good Clinical Practice, and the World Medical Association Declaration of Helsinki and its amendments. The study was approved by central or local institutional review boards, and all patients provided written informed consent.

### Outcome measures

Outcome measures included mean change from baseline in albumin-corrected serum calcium, phosphorus, and creatinine levels; estimated glomerular filtration rate (eGFR); 24-hour urinary calcium excretion; and impact of thiazide diuretics on urinary calcium. Other outcome measures included incidence of treatment-emergent adverse events (AEs) and treatment-emergent serious AEs; mean percentage change from baseline in oral calcium and calcitriol doses; and mean change from baseline in serum 1,25(OH)_2_D, bone turnover markers (BTMs), and bone mineral density (BMD). Fractional excretion of calcium (FECa), urinary calcium adjusted for body weight, and mean Ca × P product were also determined as *post hoc* analyses.

The composite efficacy outcome was the proportion of patients who achieved a reduction in the use of oral calcium supplements (≥50% reduction in dose from baseline or oral calcium dose ≤500 mg/d) and calcitriol (≥50% reduction in dose from baseline or oral calcitriol dose ≤0.25 µg/d) while normalizing or maintaining albumin-corrected serum calcium levels compared with the baseline value and not exceeding the ULN for the central laboratory [10.2 mg/dL (2.55 mmol/L)].

### Assessments

On the days of clinic visits, serum for laboratory assessments and measurement of BTMs was collected before the daily injection of study drug. Albumin-corrected serum calcium, total serum calcium, and serum phosphorus levels, and Ca × P product were assessed at baseline, week 4, week 8, every 8 weeks up to week 48, and week 52. Thereafter, total serum calcium and serum phosphorus levels, and Ca × P product were evaluated every 2 months to month 60, and albumin-corrected serum calcium levels were determined yearly. The 24-hour urinary calcium and serum creatinine levels, and eGFR were determined at baseline and at weeks 16, 32, and 52, and then every 4 months to month 60. AEs were assessed at study visits and also could be reported at any time; serious AEs were recorded within 24 hours of awareness. Serum 1,25[OH]_2_D was measured at baseline, weeks 24 and 52, and every 6 months thereafter. Serum BTMs [bone-specific alkaline phosphatase (BAP), cross-linked C-telopeptide of type 1 collagen (CTX), and aminoterminal propeptide of type 1 collagen (P1NP)] were assessed at baseline; at weeks 8, 16, 24, 40, and 52 during the first year of study; then every 4 months to month 60. BMD of the lumbar spine, total hip, femoral neck, and one-third distal radius was assessed via dual-energy x-ray absorptiometry at baseline and yearly thereafter.

Calcium and phosphorus levels were measured using commercially available assays (ADVIA^®^; Siemens Healthcare Diagnostics, Tarrytown, NY). Inorganic phosphate was measured and expressed as milligrams per deciliter of phosphorus; the standard conversion factor of 0.323 for inorganic phosphate was used to generate the data in International System of Units (mmol/L) (22). Concentrations of 1,25[OH]_2_D were determined by liquid chromatography–tandem mass spectrometry. Albumin-corrected serum calcium level was determined using the following formula: corrected calcium = serum calcium + 0.8 × (4 – serum albumin). eGFR was calculated using the Chronic Kidney Disease Epidemiology Collaboration equation (23). FECa was determined using the following formula: FECa = (24-hour urine calcium/serum calcium)/(24-hour urine creatinine/serum creatinine) (24).

BTM levels were determined using commercially available immunoassays (BAP: LIAISON^®^ BAP OSTASE^®^ immunoassay, DiaSorin, Stillwater, MN; P1NP: Cobas^®^ total P1NP, Roche Diagnostics, Indianapolis, IN; and CTX Cobas^®^*β*-CrossLaps/serum, Roche Diagnostics).

Dual-energy x-ray absorptiometry densitometers were Hologic^®^ (QDR 4500, Delphi, Horizon, or Discovery models; Hologic, Waltham, MA) or Lunar^®^ (Prodigy or iDXA models; GE Healthcare, Chicago, IL). Any changes or upgrades to the scanner or software were reviewed before use. Instrument standardization and calibration were conducted using the Bona Fide Phantom (BioClinica, Doylestown, PA). Each clinical site was required to scan the Bona Fide Phantom 10 consecutive times and submit for central analysis.

### Statistical analysis

SAS, version 9.2 (SAS Institute Inc., Cary, NC), was used for all statistical procedures. Continuous variables were summarized by descriptive statistics with the number of patients, mean, and SD; categorical variables included the number and percentage of patients. Because of the open-label nature of the study and the lack of comparator, no formal statistical testing or between-group comparisons were conducted. Data are presented as mean ± SD unless otherwise indicated.

Baseline values were defined as those measured just before initiation of rhPTH(1-84) treatment, whether that occurred at the start of REPLACE, RELAY (for patients who did not participate in or received placebo in REPLACE), or RACE (for patients who received placebo in REPLACE and did not participate in RELAY). End points for oral calcium and calcitriol levels were based on investigator-prescribed data. AEs were coded using the Medical Dictionary for Regulatory Activities, version 13.0 (https://www.meddra.org/).

## Results

### Patient disposition and baseline characteristics

Forty-nine patients who completed RELAY and/or REPLACE were enrolled from 12 US centers. The median interruption between completion of the previous study and initiation of treatment in RACE was 1.0 (interquartile range, 1.0 to 1.0) day. Forty enrolled patients (81.6%) had completed 60 months of rhPTH(1-84) treatment as of 8 May 2017. Nine patients withdrew from the study for the following reasons: patient’s decision (n = 5; none were AE-related), investigator’s decision (n = 2), AE (n = 1), or patient declined to enter the extension after the first 12 months were completed (n = 1). Table 1 summarizes key patient demographics and baseline characteristics.

**Table 1.** Patient Demographics and Baseline Characteristics

Characteristic	
No. of patients	49
Age, mean (SD), y	48.1 (9.78)
Female sex	40 (81.6)
Race	
White	46 (93.9)
Asian	2 (4.1)
Native Hawaiian/Pacific Islander	1 (2.0)
Duration of hypoparathyroidism, mean (SD), y	15.9 (12.49)
Prescribed calcitriol at baseline, µg/d	
Mean (SD)	0.67 (0.446)
≤0.25	13 (26.5)
>0.25−0.50	16 (32.7)
>0.50	20 (40.8)
Prescribed oral calcium at baseline, mg/d	
Mean (SD)	2194 (1732.3)
0−2000	31 (63.3)
>2000	18 (36.7)

Data are given as no. (%) unless otherwise indicated.

### Biochemical parameters

**Figure 2.**
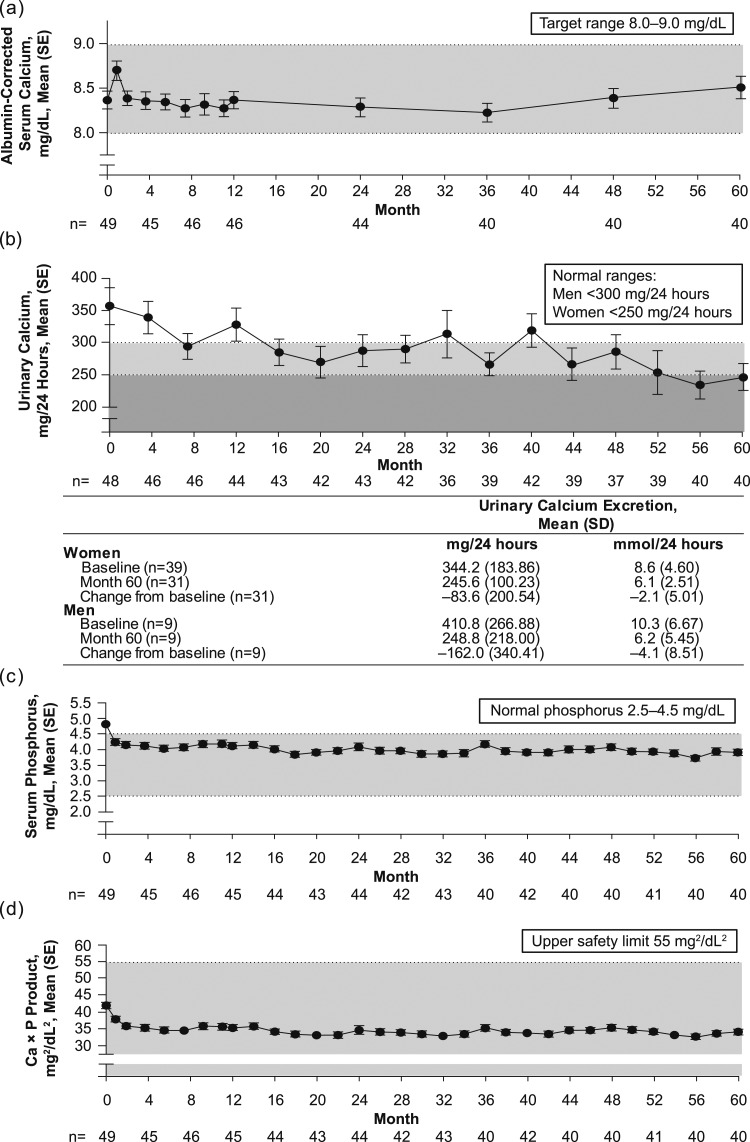
Changes in (a) albumin-corrected serum calcium level, (b) urinary calcium excretion, (c) serum phosphorus level, and (d) Ca × P product over time.

Mean albumin-corrected serum calcium concentration was maintained within the target range of 8.0 to 9.0 mg/dL (2.00 to 2.25 mmol/L) compared with baseline [mean, 8.4 ± 0.70 mg/dL (2.1 ± 0.17 mmol/L); n = 49] through month 60 [mean, 8.5±0.78 mg/dL (2.1 ± 0.20 mmol/L); n=40; Fig. 2(a)]. Mean change from baseline at month 60 was 0.10 ± 0.89 mg/dL (0.02 ± 0.22 mmol/L).

Urinary calcium excretion trended downward over 60 months of rhPTH(1-84) treatment [Fig. 2(b)]. Mean urinary calcium excretion was above normal at baseline [356.7 ± 200.37 mg/24 hours (8.9±5.01 mmol/24 hours); n = 48) and declined to a level within the normal range at month 60 [246.3 ± 132.21 mg/24 hours (6.2 ± 3.31 mmol/24 hours); n = 40). Mean change from baseline at month 60 was −101.2 ± 236.24 mg/24 hours [−2.5 ± 5.91 mmol/24 hours; 95% CI, –176.78 to –25.67 mg/24 hours (95% CI, –4.42 to –0.64 mmol/24 hours)]. Data are reported for men and women separately in Fig. 2(b). At baseline, 33 of 48 patients (68.8%) had hypercalciuria, compared with 18 of 40 patients (45.0%) at month 60. Furthermore, mean FECa was 3.1% ± 1.18% at baseline (n = 47) and decreased to 2.2% ± 1.22% at month 60 (n = 40), representing a change from baseline of –18.1% ± 47.06% (95% CI, –33.3% to –2.8%; n = 39). Mean urinary calcium normalized to body weight also declined, from 4.3 ± 2.44 mg/24 hour/kg (0.11 ± 0.061 mmol/24 hour/kg) at baseline (n = 48) to 3.0 ± 1.80 mg/24 hour/kg (0.07 ± 0.045 mmol/24 hour/kg) at month 60 [n = 40; mean change from baseline, –1.1 ± 2.62 mg/24 hour/kg (–0.03 ± 0.066 mmol/24 hour/kg)]. The mean decrease in urinary calcium was greater in the subgroup of patients who were using thiazide diuretics (n = 10) at month 60 [−177.5 ± 239.55 mg/24 hours (−4.4 ± 5.99 mmol/24 hours); 95% CI, –348.9 to –6.1 mg/24 hours (95% CI, –8.72 to –0.15 mmol/24 hours)]; however, patients who were not receiving thiazides (n = 39) also had a decrease in urinary calcium [−75.8 ± 233.59 mg/24 hours (−1.9 ± 5.84 mmol/24 hours); 95% CI, –163.0 to 11.4 mg/24 hours (95% CI, –4.1 to 0.3 mmol/24 hours); n = 30].

Serum phosphorus levels were uniformly lower than baseline throughout the 60 months of rhPTH(1-84) treatment [Fig. 2(c)]. Mean serum phosphorus concentration was 4.8 ± 0.58 mg/dL (1.56 ± 0.188 mmol/L) at baseline (n = 49) and 3.9 ± 0.66 mg/dL (1.27 ± 0.213 mmol/L) at month 60 (n = 40), with a mean change from baseline of ‒1.0 ± 0.78 mg/dL (‒0.31 ± 0.253 mmol/L). At baseline, 26 patients had serum phosphorus levels above the normal range [*i.e.,* >4.5 mg/dL (>1.45 mmol/L)] compared with only eight patients at month 60.

Mean Ca × P product was 42.1 ± 6.35 mg^2^/dL^2^ (3.4 ± 0.51 mmol^2^/L^2^; n = 49) at baseline and 34.2 ± 5.55 mg^2^/dL^2^ (2.8 ± 0.45 mmol^2^/L^2^; n = 40) at month 60. The mean change from baseline was −8.5 ± 8.29 mg^2^/dL^2^ [−0.7 ± 0.67 mmol^2^/L^2^; Fig. 2(d)].

### Renal function

**Figure 3.**
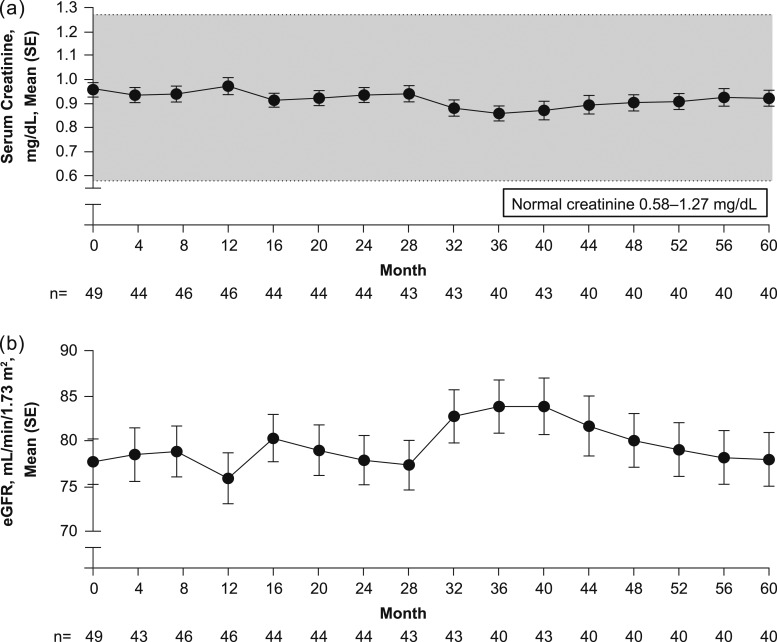
Changes in (a) serum creatinine and (b) eGFR over time.

Mean serum creatinine levels were stable with treatment over the 5 years [baseline, 0.96 ± 0.205 mg/dL (84.7 ± 18.16 µmol/L), n = 49; month 60, 0.93 ± 0.22 mg/dL (81.7 ± 19.85 µmol/L), n = 40; mean change from baseline, −0.06 ± 0.15 mg/dL (−4.9 ± 13.02 µmol/L)] and remained within the reference range throughout the study [Fig. 3(a)]. Mean eGFR by the Chronic Kidney Disease Epidemiology Collaboration equation was also stable: 77.7 ± 17.67 mL/min/1.73 m^2^ (n = 49) at baseline and 78.0 ± 18.80 mL/min/1.73 m^2^ (n = 40) at month 60 [mean change from baseline, 2.3 ± 11.81 mL/min/1.73 m^2^; n = 40; Fig. 3(b)]. A similar eGFR profile was obtained when the prespecified Cockcroft-Gault formula was used to generate the data (data not shown).

### Adverse events

No clinically significant changes in vital signs, physical examinations, or ECG parameters were observed over the treatment period. In addition, there were no clinically meaningful trends in safety laboratory results beyond those already discussed. Treatment-emergent AEs were reported in 48 of 49 patients (98.0%) while they were receiving rhPTH(1-84) treatment (Table 2). Five injection site reactions were reported in four patients (8.2%); all were mild. Severe AEs were reported in 17 patients (34.7%). AEs assessed by the investigator as related to treatment with rhPTH(1-84) were reported in 25 patients (51.0%). The most common AEs considered to be related to treatment were nausea (n = 7; 14.3%), hypercalcemia (n = 6; 12.2%), hypocalcemia (n = 4; 8.2%), tetany (n = 4; 8.2%), constipation (n = 3; 6.1%), and paresthesia (n = 3; 6.1%).

**Table 2.** Treatment-Emergent AEs Reported in ≥10% of Patients

	AE Severity, No. (%)			
Preferred Term	Mild	Moderate	Severe	All AEs (N = 49)
Hypocalcemia	8 (16.3)	10 (20.4)	0	18 (36.7)
Muscle spasms	6 (12.2)	9 (18.4)	1 (2.0)	16 (32.7)
Nausea	10 (20.4)	5 (10.2)	0	15 (30.6)
Paresthesia	12 (24.5)	3 (6.1)	0	15 (30.6)
Sinusitis	5 (10.2)	9 (18.4)	1 (2.0)	15 (30.6)
Headache	4 (8.2)	7 (14.3)	1 (2.0)	12 (24.5)
Arthralgia	1 (2.0)	9 (18.4)	2 (4.1)	12 (24.5)
Bronchitis	6 (12.2)	5 (10.2)	0	11 (22.4)
Nasopharyngitis	6 (12.2)	4 (8.2)	0	10 (20.4)
Back pain	2 (4.1)	6 (12.2)	2 (4.1)	10 (20.4)
Constipation	7 (14.3)	2 (4.1)	1 (2.0)	10 (20.4)
Diarrhea	5 (10.2)	4 (8.2)	0	9 (18.4)
Extremity pain	4 (8.2)	5 (10.2)	0	9 (18.4)
Urinary tract infection	5 (10.2)	3 (6.1)	1 (2.0)	9 (18.4)
Fatigue	2 (4.1)	4 (8.2)	2 (4.1)	8 (16.3)
Influenza	4 (8.2)	4 (8.2)	0	8 (16.3)
Vitamin D deficiency	6 (12.2)	2 (4.1)	0	8 (16.3)
Viral gastroenteritis	2 (4.1)	6 (12.2)	0	8 (16.3)
Hypercalcemia	4 (8.2)	3 (6.1)	0	7 (14.3)
Tetany	2 (4.1)	5 (10.2)	0	7 (14.3)
Upper respiratory tract infection	5 (10.2)	2 (4.1)	0	7 (14.3)
Anxiety	1 (2.0)	6 (12.2)	0	7 (14.3)
Joint sprain	3 (6.1)	3 (6.1)	1 (2.0)	7 (14.3)
Nephrolithiasis	3 (6.1)	2 (4.1)	1 (2.0)	6 (12.2)
Decreased blood calcium	5 (10.2)	1 (2.0)	0	6 (12.2)
Dizziness	5 (10.2)	1 (2.0)	0	6 (12.2)
Vomiting	4 (8.2)	2 (4.1)	0	6 (12.2)
Chest discomfort	4 (8.2)	2 (4.1)	0	6 (12.2)
Hypertension	2 (4.1)	4 (8.2)	0	6 (12.2)
Urine calcium increased	4 (8.2)	1 (2.0)	0	5 (10.2)
Myalgia	1 (2.0)	4 (8.2)	0	5 (10.2)
Dyspepsia	2 (4.1)	3 (6.1)	0	5 (10.2)
Abdominal pain	3 (6.1)	1 (2.0)	1 (2.0)	5 (10.2)
Insomnia	5 (10.2)	0	0	5 (10.2)
Muscle strain	1 (2.0)	4 (8.2)	0	5 (10.2)
Palpitations	5 (10.2)	0	0	5 (10.2)
Vitamin D decreased	3 (6.1)	2 (4.1)	0	5 (10.2)

Serious AEs occurred in 13 of 49 patients (26.5%), and none was considered by the investigator to be related to treatment with rhPTH(1-84). No cases of bone tumors or osteosarcoma were reported. One death was reported during the study. A 52-year-old woman died of acute systolic congestive heart failure thought to be related to her comorbidities (including hypertension, hyperlipidemia, and cardiomyopathy) and to be unrelated to rhPTH(1-84) treatment.

### rhPTH(1-84) and oral supplement dosing and serum 1,25(OH)_2_D level

**Figure 4.**
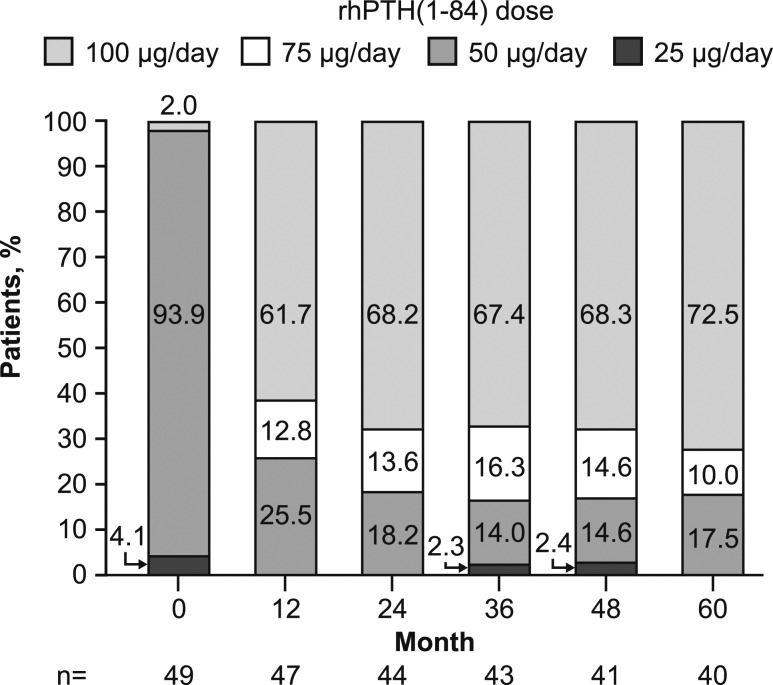
Changes in rhPTH(1-84) dosing from the starting dose for the first study visit of RACE through month 60.

**Figure 5.**
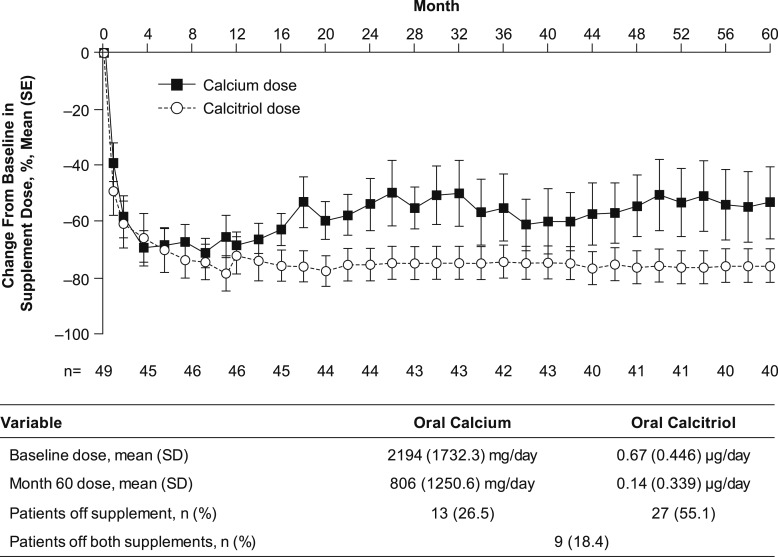
Percentage change from baseline in dose of oral calcium and calcitriol over time.

At the start of RACE, the dose of rhPTH(1-84) was 25 µg/d for 4.1% of patients (n = 2), 50 µg/d for 93.9% (n = 46), and 100 µg/d for 2.0% (n = 1; Fig. 4). At month 60, the rhPTH(1-84) dose was 100 µg/d for 72.5% of patients (n = 29), 75 µg/d for 10.0% (n = 4), and 50 µg/d for 17.5% (n = 7). Treatment with rhPTH(1-84) led to reductions in daily doses of oral calcium and calcitriol that were sustained throughout the 5-year treatment period (Fig. 5). At month 60, daily intake of supplemental oral calcium and calcitriol had declined by a mean of 53.4% ± 81.34% (95% CI, –79.4% to –27.4%) and 75.7% ± 38.44% (95% CI, –88.0% to –63.5%), respectively, from baseline. Furthermore, at month 60, 26.5% of patients (n = 13) had stopped taking oral calcium, 55.1% (n = 27) had stopped oral calcitriol, and 18.4% (n = 9) had stopped both. Despite reductions in supplementation with oral calcitriol, serum concentrations of 1,25[OH]_2_D increased by a mean of 9.1 ± 11.72 pg/mL (95% CI, 4.3 to 14.0 pg/mL; n = 25) between baseline (30.6 ± 12.21 pg/mL; n = 29) and month 60 (39.4 ± 12.66 pg/mL; n = 39).

### Composite efficacy outcome

**Figure 6.**
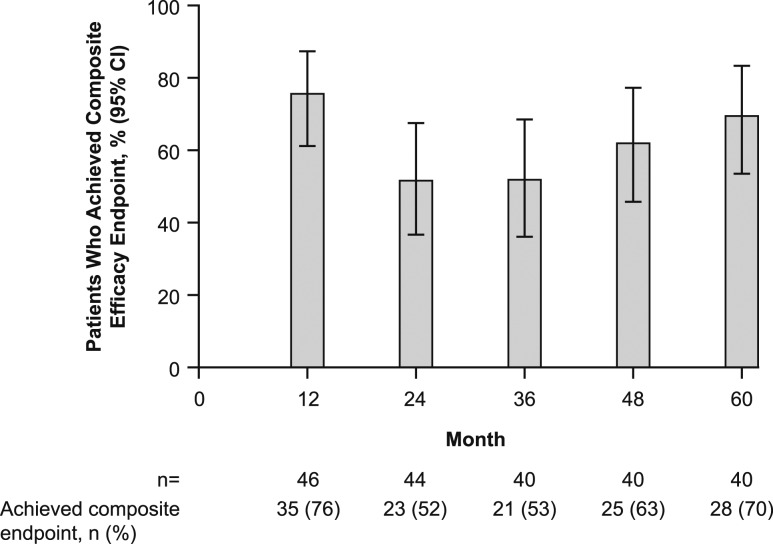
Percentage of patients who achieved the composite efficacy end point over time.

The composite efficacy outcome was achieved by 70.0% (95% CI, 53.5% to 83.4%; n = 28) of patients at month 60 (Fig. 6). Twenty-one of these patients (75.0%) received the maximum dose (100 µg/d) of rhPTH(1-84), two (7.1%) received 75 µg/d, and five (17.9%) received 50 µg/d. Of the 12 patients who did not achieve the efficacy end point, eight (66.7%) received the maximum dose, two (16.7%) received 75 µg/d, and two (16.7%) received 50 µg/d. Thus, four patients did not achieve the efficacy end point but received less than the maximum dose of rhPTH(1-84).

### Bone turnover and BMD

**Figure 7.**
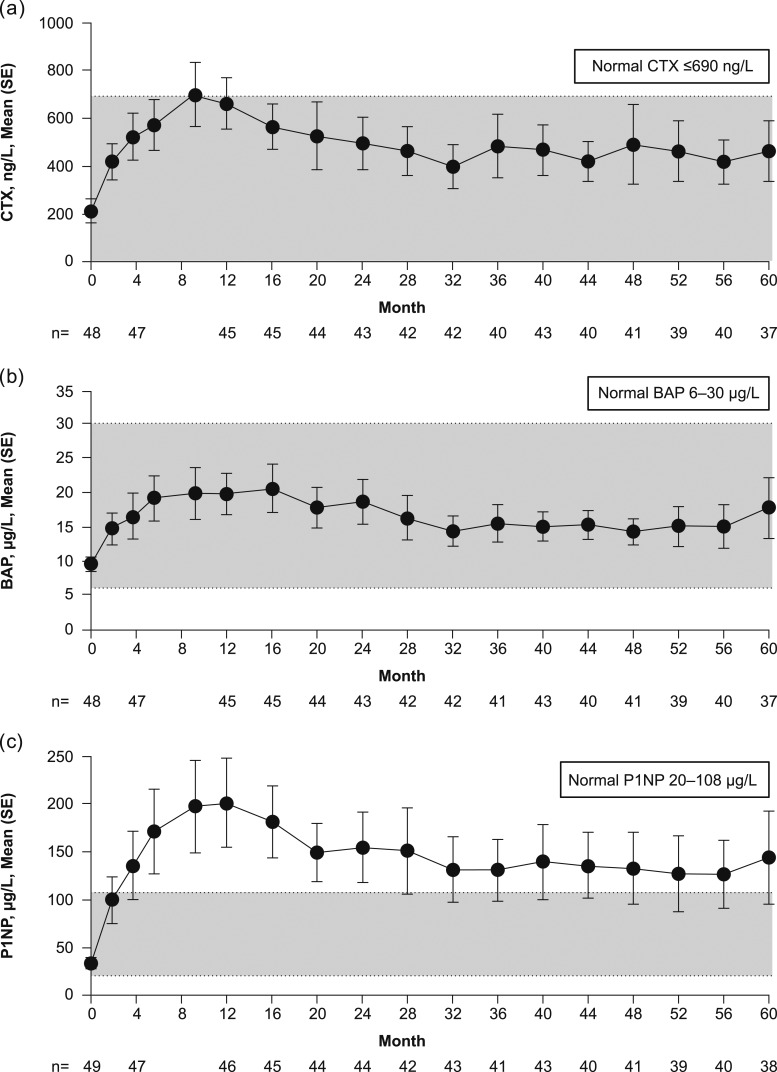
Change in serum (a) CTX, (b) BAP, and (c) P1NP BTMs over time.

Mean serum concentrations of all BTMs increased from baseline with rhPTH(1-84) treatment, reaching a plateau 6 to 16 months after initiation of treatment, then declining and remaining within normal limits for CTX and BAP, and slightly above the normal limit for P1NP (Fig. 7). Baseline, maximum, and month 60 values for BTMs are reported in Table 3. Total serum alkaline phosphatase levels, measured as part of routine safety laboratory screening, responded similarly to BAP over the course of the study (data not shown).

**Table 3.** Changes in Bone Turnover Markers

BTM	Baseline (N = 49)	Maximum Value (n = 47)^*a*^	Month 60 (n = 38)
CTX, ng/L (normal, ≤690 ng/L)	213.0 (172.34)^*b*^	699.1 (469.5)	463.7 (388.30)^*c*^
BAP, μg/L (normal, 6–30 µg/L)	9.6 (3.32)^*b*^	20.7 (12.18)^*d*^	17.8 (13.82)^*c*^
P1NP, μg/L (normal, 20–108 µg/L)	33.7 (19.72)	201.5 (160.72)^*e*^	144.1 (153.73)

Data reported as mean (SD).

^a^ Maximum value occurred at week 40 for CTX, month 12 for P1NP, and month 16 for BAP.

^b^ n = 48.

^c^ n = 37.

^d^ n = 45.

^e^ n = 46.

At baseline, mean BMD Z*-*scores at lumbar spine, total hip, and femoral neck were greater than the normal range for age, sex, and race and were particularly high at the lumbar spine (Table 4). After 60 months of rhPTH(1-84) treatment, the mean BMD Z*-*score at the distal one-third radius site was reduced compared with baseline. At other sites, mean Z*-*scores remained stable and above 1 between baseline and month 60.

**Table 4.** BMD Z*-*Score at Baseline and Month 60

	Baseline		Month 60	
Location	Mean (SD)	No.	Mean (SD)	No.
Lumbar spine	2.1 (1.47)	40	2.1 (1.52)	35
Hip, total	1.6 (1.12)	40	1.5 (1.09)	34
Hip, femoral neck	1.4 (1.24)	40	1.4 (1.27)	34
Distal one-third radius	0.9 (0.83)	40	0.5 (1.19)	35

Excludes results from six patients who were assessed by different dual-energy x-ray absorptiometry machines at baseline vs month 60 (Hologic and GE Lunar instruments, respectively).

## Discussion

Findings from 5 years of treatment with rhPTH(1-84) in the RACE study provide further evidence supporting the long-term safety and efficacy of rhPTH(1-84) in the treatment of adults with hypoparathyroidism. Along with overall reductions in oral supplementation, mean albumin-corrected serum calcium levels were maintained within the target range over 60 months of treatment, suggesting sustained long-term efficacy. Importantly, mean 24-hour urine calcium declined to levels within the normal range, and mean FECa showed an 18% decrease between baseline and 60 months, indicating that renal tubular reabsorption of calcium improved with rhPTH(1-84). Similar beneficial effects on urinary calcium excretion were seen in the only other long-term open-label study of rhPTH(1-84), the single-center HEXT (Hypo-Extended: Effect of PTH on Skeleton in Hypoparathyroidism) Study (16, 18). Interestingly, although mean 24-hour urine calcium levels declined, but remained above the ULN for women in the shorter 6-month REPLACE study, the mean urine calcium level continued to decline in this long-term study into the normal range for men and women (urinary calcium decreased by 73.6 mg/24 hours by week 24 in REPLACE compared with a decrease of 101.2 mg/24 hours by month 60 in RACE) (17). This may indicate that longer treatment may be necessary for normalization of urine calcium levels.

RACE provides additional evidence that treatment with rhPTH(1-84) improves other aspects of mineral and skeletal homeostasis. The decrease in serum phosphorus level was similar to that reported from the long-term HEXT study (18). Reductions in serum phosphorus with rhPTH(1-84) treatment likely drove the observed declines in Ca × P product concentrations, which were maintained throughout the study. In addition, kidney function was preserved; serum creatinine remained within the normal range, and eGFR was stable over 5 years in RACE.

BTMs, which are low in patients with hypoparathyroidism, increased from baseline and peaked at around 1 year after the initiation of rhPTH(1-84) treatment, then declined but remained above pretreatment values through year 5. Similar trends in the pattern of BTMs have been reported in the HEXT study (16, 18) but with differences in the magnitude of increase and time course of change that may be due to differing treatment regimens. The findings from these studies suggest the initial large increase in BTMs with rhPTH(1-84) treatment is temporary (18, 25) and is followed by a new steady state of more normal bone turnover with continued therapy. Changes in calcium and phosphorus flux from bone may at least partially explain the trend for increases in calcium supplements later in the treatment period, but this was not tested directly in this study.

BMD is often elevated in patients with longstanding hypoparathyroidism (26). In this study, no meaningful changes in BMD Z*-*scores were observed at the lumbar spine, hip (total), and hip (femoral neck), whereas a decrease in Z*-*score occurred at the distal one-third radius. These results are similar to trends seen in the HEXT trial at 6 years of follow-up (18) and are not unexpected given the differential effects of PTH on cortical and trabecular bone (27–29). Longer observations will be necessary to obtain more insights into the changes in bone density and bone quality during treatment with rhPTH(1-84).

One patient discontinued this 5-year study because of an AE, which was not considered related to treatment. The AE profile of rhPTH(1-84) was similar to that observed in the 24-week REPLACE (17) and 8-week RELAY (20) studies. The most commonly reported AEs were related to the underlying disease (*e.g.*, hypocalcemia, muscle spasms, paresthesia, nephrolithiasis) and common ailments (*e.g.*, sinusitis, nausea).

Limitations of RACE include the open-label design, the lack of a control arm, and insufficient power to perform statistical analyses. Of the 12 patients who did not achieve the three-part efficacy end point in RACE, four did not receive the maximum dose of rhPTH(1-84) of 100 µg/d. Therefore, these patients may have been undertreated. The impact of rhPTH(1-84) on health-related quality of life in adults with hypoparathyroidism was not captured in RACE. The interpretation of BMD data reported here is limited by the small sample size and the use of different densitometers with lack of standardization and cross-calibration.

Continued use of rhPTH(1-84) throughout 5 years resulted in a safety profile that was consistent with other studies; no new safety findings were identified (17, 20). Sustained improvements in mineral homeostasis and increases in BTMs were observed. Treatment with rhPTH(1-84) allowed major reductions in oral calcium supplements and active vitamin D analogs while maintaining albumin-corrected serum calcium concentration within the target range. Importantly, over the 5 years of treatment, eGFR and serum creatinine levels remained stable, mean urinary calcium excretion normalized, and serum phosphorus and Ca × P product levels improved.

## Data Availability

The datasets, including redacted study protocol, redacted statistical analysis plan, and individual participant’s data behind the results reported in this article, will be available three months after the submission of a request to researchers who provide a methodologically sound proposal after de-identification, in compliance with applicable privacy laws, data protection, and requirements for consent and anonymization. Data requests should follow the process outlined in the Data Sharing section on Shire’s website (www.shiretrials.com) and should be directed to clinicaltrialdata@shire.com. For approved requests, the researchers will be provided access to de-identified/anonymized data on a password-protected website upon Shire’s receipt of a signed Data Sharing Agreement.

## Abbreviations:

1,25[OH]_2_D: 1,25-dihydroxyvitamin D
AE: adverse event
BAP: bone-specific alkaline phosphatase
BMD: bone mineral density
BTM: bone turnover marker
Ca × P: calcium-phosphorus product
CTX: cross-linked C-telopeptide of type 1 collagen
eGFR: estimated glomerular filtration rate
FECa: fractional excretion of calcium
HEXT: Hypo-EXTended: Effect of PTH on Skeleton in Hypoparathyroidism
P1NP: aminoterminal propeptide of type 1 collagen
RACE: Open-label Study Investigating the Safety and Tolerability of NPSP558
a Recombinant Human Parathyroid Hormone [rhPTH(1-84)]: for the Treatment of Adults With Hypoparathyroidism—A Clinical Extension Study
RELAY: Study of Safety and Efficacy of rhPTH[1-84] of Fixed Doses of 25 and 50 μg in Adults With Hypoparathyroidism
REPLACE: Use of NPSP558 in the Treatment of Hypoparathyroidism; rhPTH(1-84), recombinant human PTH (1-84)
ULN: upper limit of normal
